# 晚期非小细胞肺癌*EGFR*-*TP53*共突变的研究进展

**DOI:** 10.3779/j.issn.1009-3419.2022.101.06

**Published:** 2022-03-20

**Authors:** 蓉 王, 思思 潘, 霞 宋

**Affiliations:** 1 030000 太原，山西省肿瘤医院呼吸二科 The Second Department of Respiratory, Shanxi Provincial Cancer Hospital, Taiyuan 030000, China; 2 030000 太原，山西医科大学第二临床医学院 The Second Clinical Medical College of Shanxi Medical University, Taiyuan 030000, China

**Keywords:** 肺肿瘤, TP53, 表皮生长因子受体, 共突变, 治疗, Lung neoplasms, TP53, Epidermal growth factor receptor, Co-mutation, Therapy

## Abstract

随着二代基因检测（next generation sequencing, NGS）技术的快速发展及广泛应用，多种共突变基因被我们发现。许多研究表明，共突变在表皮生长因子受体（epidermal growth factor receptor, *EGFR*）突变型非小细胞肺癌（non-small cell lung cancer, NSCLC）中对EGFR酪氨酸激酶抑制剂（EGFR tyrosine kinase inhibitor, EGFR-TKI）的反应和耐药起着重要作用。其中，*TP53*作为*EGFR*突变型NSCLC最常见的共突变基因，已被证明是一代、二代及三代EGFR-TKIs治疗预后差的重要因素，如何为*EGFR*-*TP53*共突变的晚期NSCLC患者选择最佳的治疗策略，目前仍在探索中。本文就近年来关于晚期NSCLC患者*EGFR*-*TP53*共突变的研究进展作一综述。

## Abstract (en)

With the rapid development and wide application of next generation sequencing (NGS) technology, a series of researches have revealed that concurrent genetic alterations play an important role in the response and resistance of epidermal growth factor receptor (*EGFR*)-mutant NSCLC to EGFR-tyrosine kinase inhibitor (TKI). Besides, *TP53* mutation is the most common co-mutation gene in *EGFR*-mutant NSCLC, which has been proved to confer a worse prognosis in *EGFR*-mutated patients treated with first, second and third generation of EGFR-TKIs. Currently, it is still being explored how to select the best treatment strategies for patients with concomitant presence of *TP53* mutation in *EGFR*-mutant NSCLC. Here, we review the literature on recent research progress of *TP53* concurrent mutation in *EGFR*-mutant advanced NSCLC.

表皮生长因子受体酪氨酸激酶抑制剂（epidermal growth factor receptor tyrosine kinase inhibitor, EGFR-TKI）现已成为*EGFR*突变型晚期非小细胞肺癌（non-small cell lung cancer, NSCLC）患者的一线治疗首选。然而，部分患者表现出对EGFR-TKI的原发性耐药，部分患者即便初始有反应，也存在显著的异质性结果。目前，关于EGFR-TKI原发性耐药的研究相对较少，其相关机制尚不清楚，既往研究表明，EGFR-TKI原发性耐药机制具有高度异质性，其可能的机制包括：*EGFR*敏感突变（如外显子20插入突变、T790M突变）^[1, 2]^；EGFR通路下游基因突变（如*KRAS*、*BRAF*、*PIK3CA*等）^[3, 4]^；共突变基因（如*TP53*突变、*MET*基因改变、*ALK*重排等）^[5, 6]^；microRNA水平变化^[7]^；*BIM*基因缺失多态性^[8, 9]^等。随着二代基因测序（next-generation sequencing, NGS）检测技术的发展及应用，越来越多的共突变基因被发现。众所周知，共突变在*EGFR*突变型NSCLC中对EGFR-TKI的反应和耐药起着重要作用，部分解释了异质性的结果，同时也表明了扩大基因检测范围对探索潜在耐药机制的重要性。全面的基因组分析使我们能够了解NSCLC患者的各种突变及共突变，并探索它们对临床疗效及预后的影响。本文综述了近几年国内外关于*EGFR-TP53*共突变对晚期NSCLC患者EGFR-TKI治疗及其他非TKI治疗疗效及预后影响的研究，此外，还对目前关于*EGFR-TP53*共突变人群的联合治疗策略及新型TP53靶向治疗药物的研究进展进行了小结。

## 1 抑癌基因*TP53*与肿瘤蛋白p53

*TP53*是细胞内一种重要的肿瘤抑制基因，它是包括肺腺癌、肺鳞癌在内的多种不同类型肿瘤中突变频率最高（> 50%）的基因。TP53位于人类17号染色体（17p13.1）的短臂上，作为“基因组的守护者”，它是细胞生长周期中的负调节因子，与细胞周期的调控、细胞凋亡、细胞分化、DNA修复和血管形成等重要的生物学功能有关^[10, 11]^。

抑癌基因*TP53*由11个外显子和10个内含子构成，编码的肿瘤蛋白p53由393个氨基酸构成，有3个不同的结构域：反式激活结构域、序列特异性DNA结合域（DNA-binding domain, DBD）和C末端结构域。反式激活结构域是毛细血管共济失调突变基因（ataxia telangiectasia mutated, ATM）或E3泛素蛋白连接酶鼠双微体2（mouse double minute 2, MDM2）等转录后修饰的靶点^[12]^。DBD由TP53外显子5-8编码，由102个-292个残基组成，识别与DNA修复、细胞周期阻滞或凋亡相关基因启动子中的共同序列，DBD介导的序列特异性转录活性是肿瘤蛋白p53抑瘤活性的主要机制，其中，DBD的L2环（残基163-195）和L3环（残基236-251）与锌原子结合，并在与DNA的相互作用中发挥关键作用^[13]^。最后，C末端结构域负责蛋白质的寡聚化和负调控^[14]^。

在生理状况下，p53蛋白可以通过转录依赖亦或是独立的方式发挥其抗肿瘤作用。p53蛋白作为基因组稳定性和正常细胞生理过程的“守护者”，其在调节各类型干细胞的分化、增殖和再编码方面发挥着关键作用，p53蛋白还通过直接抑制某些葡萄糖转运蛋白（glucose transporters, Gluts）的表达或间接地整合代谢相关蛋白激酶等参与了代谢调节^[15, 16]^。而细胞受到各种应激刺激，如辐射诱导的DNA损伤、化疗毒性、病毒感染、热应激、缺氧及原癌基因刺激等，都会激活p53蛋白，进而通过促进DNA修复、分化、细胞周期阻滞、衰老及凋亡等细胞活动，从而发挥其在肿瘤监测中的关键作用^[15]^。p53蛋白功能的丧失是肿瘤中常见的改变，发生的机制有多种，包括*TP53*基因突变、负调控因子MDM2^[17, 18]^对野生型p53蛋白的异常降解等。*TP53*基因突变作为许多肿瘤发生的重要原因之一，其突变类型主要为点突变和等位基因的缺失。研究^[19, 20]^发现*TP53*基因突变不仅可以导致p53蛋白正常功能的缺失，引起显性负向效应，使得基因组不稳定并下调凋亡；还可以获得新的功能，如由抑制细胞增殖转换为促进细胞生长，诱导血管内皮生长因子（vascular endothelial growth factor, VEGF）分泌，对抗肿瘤药物耐受，以及p53家族其他成员（p63和p73）的失活等，上述即被称为突变p53蛋白的“功能增益”活性（gain of function, GOF），近年来关于“GOF”的探索已成为肿瘤治疗的研究热点。

研究发现，在35%-60%的NSCLC患者中发现了*TP53*突变，且肺鳞癌的发生率高于肺腺癌（38% *vs* 12%）^[10, 21, 22]^，与其他肿瘤抑制基因不同（如*APC*、*BRCA1*或*RB1*等基因突变以截断突变为主要突变类型），*TP53*突变大多为错义突变，占总突变的75%以上^[23, 24]^。目前，随着研究的不断深入，已经发现*TP53*突变的位置和类型对预后有不同的影响，然而，现如今存在的主要问题是：缺乏对*TP53*基因突变统一的分类标准，这可能是导致关于合并TP53的预后及预测价值存在争议的原因之一。本文主要涉及到以下两种*TP53*突变的分类标准，包括根据p53蛋白结构和功能的紊乱程度，将突变分为破坏性突变和非破坏性突变。TP53的破坏性突变包括：①引入终止密码子的改变，导致p53蛋白生产的中断；②氨基酸从一种极性/电荷类别替换到另一种极性/电荷类别，以及L2或L3环内的框内缺失。非破坏性*TP53*突变包括所有未被归类为破坏性突变的突变，包括L2或L3环外的突变，如错义突变和框内缺失，以及L2或L3环内的突变，这些突变将一个氨基酸残基替换为另一个相同极性的氨基酸残基^[25, 26]^。另一种分类为根据不同位点外显子突变发生的频率将其分为位置“热点”外显子与“非热点”外显子，外显子5-8是最常见的突变位点，即“热点”外显子突变，其他突变为“非热点”外显子突变^[25, 26]^。

## 2 *EGFR*-*TP53*共突变

与标准化疗^[27-30]^相比，EGFR-TKIs已经显著改善了*EGFR*突变型NSCLC患者的生存和生活质量，一代、二代及三代EGFR-TKI具有更高的客观缓解率（objective response rate, ORR）及更长的无进展生存期（progression-free survival, PFS），现已成为晚期*EGFR*突变型NSCLC患者的一线治疗首选。然而，大约20%的患者会发生EGFR-TKI原发性耐药^[27]^。此外，即使在初始有反应的患者中，也观察到显著的异质性结果，表现为有些患者只有几周的疗效，而一些患者可能受益多年而没有出现进展。如今，随着NGS技术的发展和应用，人们发现了多种*EGFR*共突变基因。已有研究^[31-33]^表明，共突变基因可能会减弱TKI的疗效，这就部分解释了异质性患者的结果。共突变一般可以分为两类，一类是肿瘤发生早期，癌细胞具有的*EGFR*、*MET*、*TP53*等基因突变，可能导致原发性耐药；另一类则是抗肿瘤治疗过程在基因层面带来“压力选择”，使癌细胞出现更多亚克隆基因改变，与潜在的获得性耐药机制有关。其中*TP53*是*EGFR*突变NSCLC患者中最常见的共突变基因，占55%-65%^[31, 34]^。

## 3 *EGFR*-*TP53*共突变对NSCLC治疗的影响

### 3.1 *EGFR-TP53*共突变对EGFR-TKI治疗的影响

#### 3.1.1 不区分*EGFR-TP53*突变亚型

Kim等^[34]^的一项回顾性研究通过对两个队列的基因图谱分析，探讨了共同发生的基因突变分别对一/二代EGFR-TKI及三代EGFR-TKI治疗晚期NSCLC疗效的预测作用。队列1中，*TP53*突变的频率最高，与TP53野生型相比，*TP53*共突变有缩短PFS的趋势（mPFS：12.5个月*vs* 14.7个月，*P*=0.087），在调整了年龄、性别、吸烟状况、美国东部肿瘤协作组（Eastern Cooperative Oncology Group, ECOG）评分和EGFR亚型后，多变量分析进一步证实了*TP53*突变预示着较短的PFS（HR=2.0, 95%CI: 1.0-3.93, *P*=0.038）和更差的总生存期（overall survival, OS）（HR=3.6, 95%CI: 1.5-8.77, *P*=0.004），提示对于接受一/二代EGFR-TKI治疗的患者来说，合并*TP53*突变是一个不良预后因素；队列2的结果提示：*TP53*共突变是在一/二代EGFR-TKI耐药后出现T790M阳性且接受三代EGFR-TKI治疗患者中一个独立不良预后因素。Xu等^[35]^的研究选择了具有*EGFR*基因外显子19的框架内缺失（19del）与第858位的外显子21的点突变（L858R突变）的晚期NSCLC患者，根据其接受一代EGFR-TKI治疗后的PFS长短将其分为两组（PFS≤6个月组，PFS≥24个月组），在治疗前和复发后（或最后一次随访）对其肿瘤样本进行了416个癌症相关基因的NGS测序，他们发现，在PFS≤6个月的患者中，*TP53*突变的发生率很高（88% *vs* 13%, *P* < 0.001），同样提示*TP53*共突变可能是EGFR-TKI治疗晚期*EGFR*突变型NSCLC预后不良的因素之一。Canale等^[36]^在既往研究的基础上进一步证实了在接受一代、二代EGFR-TKI一线治疗的*EGFR*突变型NSCLC中，*TP53*外显子8突变是其不良预后因素，此外，研究者们又进一步探讨了*TP53*共突变在预测一代/二代EGFR-TKI一线治疗后进展出现T790M突变且接受三代EGFR-TKI治疗的患者预后中的作用，他们对符合条件的*EGFR*-T790M突变患者进行了亚组分析，发现*TP53*外显子8突变组有着更差的PFS（HR=2.39, 95%CI: 0.77-7.45, *P*=0.134）和OS（HR=4.86, 95%CI: 1.25-18.90, *P*=0.023），尽管可能由于样本量小的缘故，差异并不显著，但仍提示*TP53*外显子8突变可能对三代EGFR-TKI疗效差也有一定的预测作用。

Zhang等^[37]^的荟萃分析纳入了8项研究，共计2, 979例患者，结果提示：与*TP53*野生型相比，*TP53*突变型患者有着较差的OS（HR=1.73, 95%CI: 1.22-2.44, *P*=0.002），进一步根据是否接受靶向治疗进行了亚组分析，同样提示，在接受EGFR-TKI治疗的患者中，*TP53*突变是OS和PFS的不良预后因素（OS: HR=2.29, 95%CI: 1.39-3.76, *P*=0.001; PFS: HR=2.18, 95%CI: 1.42-3.36, *P* < 0.001）。然而，可能由于有限的数据，该分析并未发现*TP53*突变亚型（非破坏性突变、错义突变）对预后影响的阳性结果。Qin等^[38]^的荟萃分析纳入15项研究，共计1, 342例患者，整体而言，合并*TP53*突变的分子特征与较短的PFS和OS相关（PFS: HR=1.88, 95%CI: 1.59-2.23, *P* < 0.001; OS: HR=1.92, 95%CI: 1.55-2.38, *P* < 0.001），且基于靶向治疗类型的亚组分析显示：合并*TP53*突变与接受EGFR-TKI或ALK-TKI治疗患者较差的预后有关，尤其在一线接受EGFR-TKI治疗的患者中，合并*TP53*突变患者的预后更差（PFS: HR=1.69, 95%CI: 1.25-2.27, *P* < 0.001; OS: HR=1.94, 95%CI: 1.36-2.76, *P* < 0.001）。来自我国学者的一项真实世界的数据分析^[39]^进一步验证了以上的结论，*EGFR-TP53*共突变的NSCLC患者有着更短的生存和预后。综合以上研究，均提示我们*TP53*共突变是接受一代、二代、三代EGFR-TKI治疗NSCLC患者预后差的重要影响因素之一，其中*TP53*外显子8突变可能与对EGFR-TKI治疗特别不敏感有关。

#### 3.1.2 区分*EGFR-TP53*突变亚型

目前，根据已有的研究结果，我们知道并非所有类型的*EGFR*突变和*TP53*突变都对EGFR-TKI治疗的生存及预后有影响，通过突变亚型来考虑TKI类药物对晚期NSCLC生存及预后的影响无疑是十分明智的。

Jiao等^[40]^的研究基于癌症基因图谱数据库探讨了*TP53*突变以及不同位点外显子突变在晚期NSCLC中的预后价值，共收集了1, 441例NSCLC患者数据。该研究首次根据突变位点不同将*TP53*突变分为四组：分别为A组：*TP53*野生型；B组：*TP53*外显子2、3、10突变（少见突变组）；C组：*TP53*外显子5、7、8、9突变（预后良好的突变组）；D组：*TP53*外显子4、6，多个外显子突变及1个未知外显子突变（预后不良的突变组）。这四组的OS分别为27个月、未达到、21个月和13个月，差异具有统计学意义（*P* < 0.001）。OS的数据结果提示D组突变位点患者的预后较B组差。该研究又分析了*EGFR*突变类型与发生*TP53*共突变频率之间的关系，发现*EGFR*突变型患者的*TP53*突变率较高，其中*EGFR*外显子19/21突变型较非外显子19/21突变型的*TP53*突变率高（66.2% *vs* 62.7%, *P* < 0.001），进一步结合*EGFR*突变类型（野生型、外显子19/21突变型、非外显子19/21突变型）与*TP53*突变类型（野生型、预后良好突变型和预后不良突变型）将患者分为9组，分别为野生-野生、野生-好、野生-差、外显子19/21-野生、外显子19/21-好、外显子19/21-差、非外显子19/21-野生、非外显子19/21-好和非外显子19/21-差，这9组的中位OS分别为27个月、18个月、12个月、未达到、未达到、21个月、未达到、16个月和21个月，差异有统计学意义（*P* < 0.001）。结果提示在不同类型的*EGFR*突变型患者中，呈现出这样的趋势：*TP53*野生型的患者预后最好，*TP53*外显子5、7、8、9突变的患者预后中等，*TP53*外显子4、6，多个外显子突变及未知突变的患者预后最差。然而令人奇怪的是，在*EGFR*非外显子19/21突变的患者中，与总体趋势不同，*TP53*外显子5、7、8、9突变的患者似乎预后最差，这种异常现象可能与非外显子19/21突变组中存在的几种生物学和遗传学亚型有关。Jiao等^[40]^的研究表明*TP53*突变可作为晚期NSCLC的不良预后因素，且不同位点外显子的突变对临床预后的影响不同，因此，在未来的临床诊疗工作中，对*EGFR-TP53*共突变进行细化分型对于评估NSCLC患者的预后有重要的临床价值。

Hou等^[41]^进行了一项在中国晚期或复发NSCLC患者中观察*TP53*共突变对一代EGFR-TKI疗效影响的研究，纳入了71例接受EGFR-TKI治疗的*EGFR*阳性晚期NSCLC患者，其中*TP53*共突变有43例（60.6%），相较于*TP53*野生型患者，*TP53*突变型的患者有着显著更短的中位PFS和OS（mPFS：6.5个月*vs* 14.0个月，*P*=0.025；mOS：28.0个月*vs* 52.0个月，*P*=0.023），而且*TP53*突变型患者的疾病控制率（disease control rate, DCR）和ORR也均低于*TP53*野生型患者（DCR: 76.7% *vs* 89.3%, *P*=0.160; ORR: 25% *vs* 28%, *P*=0.374），此外，在对*TP53*突变型患者的亚组分析中，发现*TP53*非错义突变、非破坏性突变，以及外显子6、7和非DBD区突变的NSCLC患者有着更差的预后，该研究提示*TP53*共突变降低了*EGFR*突变型晚期NSCLC对EGFR-TKI的反应性，其中*TP53*非错义突变、非破坏性突变，以及外显子6、7和非DBD区的突变是提示晚期NSCLC患者接受一代EGFR-TKI治疗预后不良的独立预测因素。然而Labbe等^[42]^的一项研究纳入了105例*EGFR*突变型NSCLC患者，其中43例合并*TP53*突变，32例*TP53*突变为错义突变，该研究评估了*TP53*突变状态对接受EGFR-TKI治疗患者预后的影响，结果显示：接受EGFR-TKI治疗的*TP53*突变型（*n*=24）患者的PFS相较于TP53野生型（*n*=36）缩短了6.8个月（10个月*vs* 16.8个月，HR=1.74，95%CI：0.98-3.10，*P*=0.06），但差异并没有统计学意义；当限制*TP53*突变为错义突变时，分析发现合并*TP53*错义突变（*n*=17）患者的PFS相较于*TP53*野生型（*n*=36）显著缩短（HR=1.91, 95%CI: 1.01-3.60, *P*=0.04）。该研究结果表明携带*TP53*错义突变的NSCLC患者，而不是非错义突变，其接受EGFR-TKI治疗的PFS显著缩短，这两项研究有相矛盾的地方，我们认为与这两项研究的样本量较少有关系，未来有必要进行更大规模的队列分析，以进一步研究TP53的细化分型及分类对预后的影响。

Liu等^[43]^首次采用两种分类系统（基于突变的位置和类型）进行比较*TP53*共突变对预后的价值，且均在预后不良组中发现了*TP53*外显子8的存在，表明发生在外显子8位点上的突变可以作为晚期NSCLC的预后生物标志物，更进一步发现在接受三代EGFR-TKI治疗的患者中，*TP53*突变的潜在预后价值只存在于*EGFR* 19del突变的患者中。此外，对于*TP53*外显子8突变对预后的影响，Canale等^[36]^同样也发现*TP53*外显子8突变与DCR显著降低和OS缩短有关，但数据仅在*EGFR* 19del突变的患者中有差异。

Yu等^[44]^进行了一项开放、单臂、前瞻性、多中心的Ⅱ期临床试验，对180例*EGFR*突变型患者的血浆循环肿瘤DNA（circulating tumor DNA, ctDNA）进行了NGS分析，其中115例（63.9%）携带*TP53*突变，秉承既往研究^[25, 26]^对TP53分型的定义，进一步对*TP53*突变进行了细化分型，53例患者（46.1%）存在*TP53*破坏性突变，62例（53.9%）存在*TP53*非破坏性突变。研究分析了在不同*EGFR*突变亚型分组中*TP53*突变对EGFR-TKI临床疗效的影响，发现*EGFR*外显子19-*TP53*突变患者的DCR和ORR均高于*EGFR* L858R-*TP53*突变患者（DCR: 80.00% *vs* 58.33%; ORR: 94.55% *vs* 86.67%），且在*EGFR*外显子19突变亚群中，*TP53*“热点”外显子突变患者的ORR（94.5%）和DCR（80.0%）较高，*TP53*非破坏性突变患者的ORR（87.1%）和DCR（67.0%）最低。进一步对*TP53*突变对EGFR-TKI预后价值进行了分析，*TP53*突变型患者的OS和PFS均显著低于*TP53*野生型患者（OS：21.2个月*vs* 32.0个月，*P* < 0.001，HR=0.54；PFS：8.4个月*vs* 12.81个月，*P*=0.007，HR=0.66），在*TP53*突变亚群中，根据*EGFR*不同突变状态进行分组，发现*EGFR* 19-*TP53*突变的患者比*EGFR* L585R-*TP53*突变的患者有更长的PFS和OS（PFS：9.47个月*vs* 7.17个月，HR=1.532；OS：22.6个月*vs* 17.2个月，*P*=0.369，HR=0.769），因大多数*TP53*突变是在外显子5-8发现的，该研究又比较了*TP53*外显子5-8突变患者的预后差异，结果提示：与野生型*TP53*相比，*TP53*外显子6、7的突变与较差的PFS和OS显著相关。随后，研究又分析了*TP53*的破坏性/非破坏性突变与预后之间的关系，发现与野生型患者相比，*TP53*破坏性和非破坏性突变患者的OS和PFS均显著缩短，然而，破坏型和非破坏型患者的生存差异却无统计学意义，进一步联合*TP53*突变类型和不同外显子突变对患者进行生存分析，发现在*TP53*非破坏性突变患者中外显子5突变患者的OS最高，然而在*TP53*破坏性突变患者中外显子5突变患者的OS较低（24.10个月*vs* 14.47个月，*P* < 0.05，HR=2.04）。因此，*TP53*破坏性突变和外显子5突变可以被认为是影响患者预后的一个因素。

以上研究均表明，*TP53*突变的位置和类型对预后有不同的影响，作为一种热门的预后指标，目前关于*TP53*破坏性突变及非破坏性突变对预后的影响存在争议，我们期待未来有更大规模的队列分析，以进一步研究TP53的细化分型及分类对预后的影响。

### 3.2 *EGFR-TP53*共突变对非EGFR-TKI治疗的影响

有一项研究^[42]^对105例*EGFR*突变NSCLC进行组织NGS或Sanger测序，发现105例患者中有43例（41%）存在*EGFR-TP53*共突变，43例共突变患者中，32例（74%）*TP53*突变为错义突变。研究评估了*TP53*突变状态对预后的影响，发现在76例早期接受手术切除的患者中，*TP53*突变型及野生型的PFS与OS均无显著差异（PFS: HR=0.99, 95%CI: 0.56-1.75, *P*=0.96; OS: HR=1.39, 95%CI: 0.70-2.77, *P*=0.35）；在接受辅助化疗的患者中（*n*=34），15例突变型，19例野生型，PFS（HR=1.52, 95%CI: 0.75-3.09, *P*=0.25）与OS（HR=1.79, 95%CI: 0.80-4.03, *P*=0.16）均不受*TP53*突变的影响。Wang等^[45]^进行了一项关于*TP53*共突变对免疫检查点抑制剂（immune checkpoint inhibitors, ICIs）疗效影响的研究，该研究基于“cBioPortal”数据库纳入了240例接受免疫治疗的NSCLC患者，其中206例患者接受抗程序性死亡受体1/程序性死亡受体配体1（programmed death-1/programmed death-ligand-1, PD1/PD-L1）单一治疗，34例患者接受的是PD-1/PD-L1抑制剂联合细胞毒性T淋巴细胞相关抗原4（cytotoxic T lymphocyte-associated antigen 4, CTLA-4）抑制剂的双免治疗方案。结果显示：在PD-1/PD-L1抑制剂单药治疗队列中，*TP53*突变型NSCLC患者的PFS显著优于*TP53*野生型患者（4.3个月*vs* 2.5个月，*P*=0.001, 9），同样在双免联合治疗队列中观察到了*TP53*突变型NSCLC的PFS获益趋势（6.3个月*vs* 5.4个月，*P*=0.12），但差异并没有统计学意义。这表明在整个队列中，相较于*TP53*野生型患者，*TP53*突变型NSCLC患者接受免疫治疗均可以获得更长的PFS，这与Assoun等^[46]^报道的*TP53*突变型NSCLC患者的mPFS显著延长（4.5个月*vs* 1.4个月，*P*=0.03）的结果相一致。同时，该研究又根据*TP53*共突变的基因类型进行了亚组分析，结果显示：在*EGFR-TP53*共突变亚组中，与*EGFR*野生型-*TP53*野生型患者相比，*EGFR*野生型-*TP53*突变型患者的PFS显著延长（4.3个月*vs* 2.5个月，*P*=0.001），然而*EGFR*突变型-*TP53*突变型患者的PFS却没有显著改善（3.4个月*vs* 2.5个月，*P*=0.707）。结合已有研究^[47]^报道的*EGFR*突变与ICIs的疗效差相关，这似乎说明*EGFR*突变的“负性效应”可能会部分抵消*TP53*突变对ICIs疗效的“正面效应”，可能是*EGFR-TP53*共突变NSCLC患者免疫治疗疗效差的原因之一。因此，近年来，以ICIs为主的免疫疗法已成为NSCLC治疗中不可或缺的部分，关于寻找预测其疗效特异生物标志物的研究更是如火如荼地进行，已有不少重要发现，未来我们期待对更多的患者开展研究，进一步探索不同的共突变模式对ICIs疗效的影响。

## 4 *EGFR-TP53*共突变的治疗策略

### 4.1 以EGFR-TKI为核心的联合疗法

#### 4.1.1 EGFR-TKI+抗血管生成治疗

RELAY研究^[48]^是一项探索雷莫芦单抗联合厄洛替尼治疗EGFR阳性NSCLC患者疗效及预后的全球多中心、随机、双盲、安慰剂对照的Ⅲ期研究，入组未经治疗的*EGFR*突变晚期NSCLC患者（突变类型限于外显子19del及外显子21L858R），按1:1随机分入试验组（雷莫芦单抗+厄洛替尼）和对照组（安慰剂+厄洛替尼）。该研究基线测序显示43%的患者合并*TP53*突变，同样发现*TP53*是最常见的共突变之一，且无论是外显子19del还是外显子21L858R突变患者，基线合并*TP53*共突变的患者接受联合治疗的PFS均优于对照组（*EGFR*外显子19del-*TP53*突变组：18个月*vs* 9.9个月，HR=0.50，95%CI：0.29-0.85；*EGFR*外显子21L858R-*TP53*突变组：14.7个月*vs* 10.8个月，HR=0.56，95%CI：0.34-0.95），该研究亦证实加入抗血管生成治疗之后，部分疗效、预后较差的患者也可以显著获益，提示TKIs联合抗血管生成治疗可能作为*EGFR*敏感突变合并*TP53*突变人群的优选治疗策略之一。此外，我国学者牵头开展的使用吉非替尼联合小分子抗血管生成药物阿帕替尼一线治疗的ACTIVE研究^[49]^是一项多中心、双盲、安慰剂对照、随机的Ⅲ期研究，试验组和对照组分别有73例（46.5%）和72例（46.8%），患者有基线*TP53* NGS检测数据。*TP53*突变和*TP53*外显子8突变的患者在*EGFR*外显子19缺失的患者中分别占43.0%（34/79）和16.5%（13/79）；在*EGFR*外显子21L858R患者中分别占54.5%（36/66）和7.6%（5/66）。分析显示，*TP53*突变的患者接受吉非替尼联合阿帕替尼治疗的PFS有获益趋势（mPFS：12.5个月*vs* 10.1个月，HR=0.56，95%CI：0.31-1.01；*P*=0.051, 6），此外，该研究还发现*TP53*外显子8突变的患者采用吉非替尼联合阿帕替尼治疗的PFS获益更加显著（mPFS：15.7个月*vs* 11.9个月，HR=0.24，95%CI：0.06-0.91；*P*=0.024, 1），研究表明*EGFR-TP53*共突变患者更适合联合治疗，特别是*TP53*外显子8突变患者联合治疗获益更大。

#### 4.1.2 EGFR-TKI+化疗

Yang等^[50]^的一项回顾性研究探讨了EGFR-TKI联合化疗相较于EGFR-TKI单药治疗能否使*EGFR*突变型NSCLC患者受益，同时分析了*TP53*共突变的分子特征对其治疗方案的影响。该研究纳入了137例*EGFR*突变型晚期肺腺癌患者，研究人群均在一线治疗前接受了NGS检测，其中75例患者合并了*TP53*突变。结果显示：在96例接受EGFR-TKI单药治疗的患者中，合并*TP53*突变的患者有着显著缩短的PFS（11.4个月*vs* 16.6个月，*P*=0.003），在联合治疗组中，无论是否合并*TP53*突变，其PFS结果相差不大（18.9个月*vs* 19.1个月，*P*=0.552）。在合并*TP53*突变的患者中，相较于单药治疗，联合治疗的PFS获益更为显著（合并*TP53*突变：11.4个月*vs* 19.1个月，*P*=0.001，HR=0.407；未合并*TP53*突变：16.6个月*vs* 18.9个月，*P*=0.379，HR=0.706）。此外，该研究进一步根据*TP53*突变位点不同将其分为两组：A组：外显子4、6位点突变（*n*=20，可能预后差的突变类型）；B组：非外显子4、6位点突变（*n*=55，可能预后良好的突变类型），分析显示，在单药治疗组，A组的PFS显著低于B组（8.0个月*vs* 12.7个月，*P*=0.045）；在联合治疗组，两组均可从联合治疗中获益，但A组的获益更为显著（A组：8.0个月*vs* 21.0个月，*P*=0.004，HR=0.181；B组：12.7个月*vs* 18.3个月，*P*=0.044，HR=0.555），该研究提示，在*EGFR-TP53*共突变人群中，EGFR-TKI单药治疗疗效不佳，EGFR-TKI联合化疗的治疗方案可能使患者受益更多，目前关于此方面研究仍较少，我们期待未来有更多的研究进一步验证EGFR-TKI联合化疗在*EGFR-TP53*共突变人群中的获益趋势，为我们的临床决策提供更加充分的循证医学证据。

### 4.2 新型TP53通路靶向治疗

在以EGFR-TKI为核心的联合治疗的基础上，近年来，专门靶向*TP53*突变的新型药物也有一定进展。p53蛋白会因基因突变、缺失或者异常降解，导致p53抑制肿瘤功能的丧失，这已得到大家公认，在一些肿瘤细胞中E3泛素蛋白连接酶MDM2过度表达，与p53蛋白结合并促进其泛素化降解，直接降低p53蛋白的稳定性和活性，帮助肿瘤逃避打击^[51]^。基于此，以MDM2-p53为靶点设计开发全新机制的抗肿瘤药物，是当下全球肿瘤药物研发领域热点与重点之一^[52]^。此外，MDM2还具有独立于p53的致癌活性，针对靶向MDM2的药物也在研究开发中^[53]^，目前，仍没有批准针对靶向MDM2的治疗药物，MDM2的相关信号通路在肿瘤免疫学中仍知之甚少。因此，Zhou等^[54]^的一项研究一方面揭示了MDM2调控CD8^+^T细胞中的信号传导子和转录活化子5（signal transducers and activators of transcription 5, STAT5）的稳定性，这对于有效的抗肿瘤免疫至关重要；另一方面，从临床的角度，靶向p53和MDM2相互作用小分子药物APG115，可诱导CD8^+^T细胞中的MDM2表达，稳定STAT5，增强T细胞功能，并与免疫疗法协同作用，诱导抗肿瘤免疫，且这类药物的疗效不依赖于肿瘤p53状态，目前APG-115已经进入临床试用。

Hsiue等^[55]^利用内源性抗原的呈递机制，鉴定了肿瘤细胞表面的p53抗原肽-主要组织相容性复合体（major histocompatibility complex, MHC）复合物，并利用该复合物成功研制出了一种靶向*TP53*基因常见突变的双特异性抗体，此抗体的一端与肿瘤细胞结合，另一端以肿瘤选择性的方式触发T细胞的细胞毒作用和细胞因子的产生，并证实了该双特异性抗体可以有效识别含有突变型*p53*的肿瘤细胞，激活T细胞以发挥抗肿瘤效应，鉴于这种双特异性抗体可以靶向传统方法难以瞄准的肿瘤细胞内抗原，势必会在未来揭开多种抑癌基因驱动肿瘤的治疗新篇章，再者，我们已经发现了*TP53*突变的“GOF”活性，即部分*TP53*突变会产生突变体，该突变体不仅会抑制剩余野生型P53蛋白的功能，同时自身还拥有新的功能，我们称之为“功能获得性突变”，不仅可激活雷帕霉素靶蛋白（mammalian target of rapamycin, mTOR）信号通路而促进肿瘤细胞的存活，还可激活细胞迁移相关的信号通路，来促进癌症的侵袭和转移。因此，靶向*TP53*突变的“功能获得性突变”也有望为治疗携带*TP53*突变肿瘤的药物研发提供一个新的视角^[56]^。

目前TKI类药物已经成为*EGFR*阳性晚期NSCLC患者一线治疗的优选方案，但大多数患者仍会出现耐药，进一步破解耐药机制无疑是提升靶向治疗效果的关键，其中一部分耐药机制就包括共突变，超过90%的*EGFR*阳性患者存在共突变，且共突变的种类和数量对治疗应答和耐药机制有显著影响。基于本文所述的临床证据，*TP53*共突变尤其是外显子8突变对EGFR-TKI的疗效有明显影响，而以EGFR-TKI为核心的联合治疗可能更适合这类患者，目前，临床上针对其的治疗已不局限于EGFR-TKI单药治疗，EGFR-TKI联合抗血管生成治疗或化疗已经显示出对*EGFR-TP53*共突变的人群有着更佳的临床获益。但需要精准识别共突变类型和个数，以实现真正意义上的个体化治疗，未来更需要专门针对共突变患者开展临床研究。另一方面，到目前为止还没有专门针对NSCLC *TP53*突变的药物上市，但已有药物进入临床试用，我们期待其有令人满意的临床价值，为治疗*EGFR-TP53*突变型NSCLC患者提供一个新的视角。

## 5 小结

近年来，随着NGS技术的快速发展及广泛应用，越来越多的*EGFR*共存基因突变被我们发现，许多研究也已经表明共突变在*EGFR*突变型NSCLC中对EGFR-TKI的反应及耐药起着重要作用，其中，*EGFR-TP53*共突变作为晚期NSCLC中最常见的共突变类型，迫切需要了解*EGFR-TP53*共突变与EGFR-TKI疗效及预后之间的关系，指导临床医生为这部分患者选择最佳的治疗策略。本文描述了多项关于*TP53*共突变在*EGFR*突变型NSCLC患者中的研究，提示*TP53*突变的位置和具体突变类型对EGFR-TKI疗效、生存及预后有着不同的影响。针对*EGFR-TP53*共突变晚期NSCLC的治疗策略选择，尚且没有规范化指南对其进行指导，通过本文所述研究不难发现，EGFR-TKI联合疗法可能对*EGFR-TP53*共突变的人群有着更佳的临床获益。而且，令人期待的是，对于靶向*TP53*突变的药物研发，可能是一种有潜力的*TP53*突变肿瘤的特异性治疗策略。未来更需要专门针对*EGFR-TP53*共突变NSCLC患者开展临床研究，以实现更加精准的个体化治疗。
